# NPY^+^-, but not PV^+^-GABAergic neurons mediated long-range inhibition from infra- to prelimbic cortex

**DOI:** 10.1038/tp.2016.7

**Published:** 2016-02-16

**Authors:** R Saffari, Z Teng, M Zhang, M Kravchenko, C Hohoff, O Ambrée, W Zhang

**Affiliations:** 1 Laboratory of Molecular Psychiatry, Department of Psychiatry, University of Münster, Münster, Germany

## Abstract

Anxiety disorders are thought to reflect deficits in the regulation of fear memories. While the amygdala has long been considered a site of storage of fear memories, newer findings suggest that the prefrontal cortex (PFC) is essential in the regulation of amygdala-dependent memories and fear expression. Here, activation of the prelimbic cortex (PrL) enhances the expression of fear, while an elevated activity in the infralimbic cortex (IL) enhances fear extinction. Despite the presence of these facts, we still know very little about the synaptic interconnectivity within the PFC. The aim of the present study was to investigate the inhibitory circuits between prelimbic and IL using morphological and electrophysiological methods. Our immunohistochemical analysis revealed that the distribution of PV^+^- and NPY^+^-GABAergic neurons was strikingly different within the PFC. In addition, we provided the first experimental evidence that the pyramidal neurons in the PrL received a direct inhibitory input mediated by bipolar NPY^+^-GABAergic projection neurons in the IL. Deletion of the anxiety-related neuroligin 2 gene caused a decrease of this direct synaptic inhibition that originated from the IL. Thus, our data suggested that activation of the IL might not only directly activate the corresponding downstream anxiolytic pathway, but also suppress the PrL-related anxiogenic pathway and thus could differentially bias the regulation of fear expression and extinction.

## Introduction

Emotional memories and regulation of these are important for guiding adaptive behavior. Mental disorders, such as anxiety disorders including panic disorder and post-traumatic stress disorder, are thought to reflect deficits in regulation of emotional memories.^1^ While the amygdala has long been considered a site of storage of emotional memories, the prefrontal cortex (PFC) with its extensive connections to subcortical limbic areas and thalamus^2, 3^ has been suggested to be essential in the regulation of amygdala-dependent memories and fear expression, especially following extinction.^4, 5, 6^ Damages in the PFC have been found to lead to dramatic alterations of the capacity of mammals to cope emotionally with environmental changes, pointing to the great importance of the PFC for the regulation of emotional reactions.^7^

Within the PFC, the dorsally located prelimbic cortex (PrL) projects primarily to the basal amygdala nucleus^2, 8, 9^ that is critical for the expression of conditioned fear.^10, 11^ On the other hand, the infralimbic cortex (IL) in the ventral part of the PFC contributes the majority of PFC inputs to the central nucleus of the amygdala^12, 13^ that plays a key role in the expression of fear extinction.^14, 15, 16^ Thus, published data make it very clear that the PFC is not functionally monolithic, but that there exists a dorsal–ventral functional dichotomy, such that the activation of the PrL drives and enhances the expression of fear, while an elevated activity in the IL suppresses and terminates these behaviors after extinction.^1, 16, 17^ Successful extinction requires the activation of an intact IL, which suppresses conditioned increases in amygdala activity, and subsequently reduces fear responses. Consequently, failure to retrieve extinction, as may occur in diseases like panic disorder and post-traumatic stress disorder, is thought to reflect a lack of IL-mediated suppression of amygdala activity, leading to persistent fear responses.^17^ Together, these data strongly suggest that the dichotomic circuit between IL and PrL represents a common node in the central regulation circuits that bi-directionally modulates the fear expression.^1, 16, 17^

In the cerebral cortex, the diversity of GABAergic interneurons is manifested by their different morphological, electrophysiological and neurochemical features. So far, over 20 different subtypes of GABAergic interneurons have been classified based on the specific proteins they express.^18, 19, 20, 21^ In particular, the calcium-binding protein parvalbumin (PV) is a crucial marker in defining the most predominant interneuron subtype within the cerebral cortex,^18, 21, 22^ which comprises ~40% of the total GABAergic cortical interneuron population.^23^ Neuropeptide Y (NPY) has been shown to be important in the modulation of anxiety.^24, 25^ NPY-expressing-neurons are less abundant, but widely distributed throughout the depth of the cortex and are more frequent in layers II–III and VI.^26^ Despite the existence of many data about the GABAergic interneurons in the cerebral cortex, there are few detailed studies examining the GABAergic inhibitory neurons in the PFC.^22, 27, 28^

Neuroligins are proteins belonging to a family of postsynaptic cell adhesion molecules that are expressed ubiquitously in the brain.^29^ They are differentially localized with respect to the postsynaptic specializations of excitatory and inhibitory synapses.^30, 31, 32^ One member of the neuroligin family, neuroligin 2 (Nlgn2) is preferentially localized in inhibitory synapses,^31^ and determines and fine-tunes the function of central inhibitory synapses.^33, 34, 35, 36, 37^ Our previous data demonstrated that deletion of the corresponding gene *Nlgn2* in mice perturbs GABAergic and glycinergic synaptic transmission and leads to a loss of postsynaptic specializations specifically at perisomatic inhibitory synapses.^34^ Furthermore, *Nlgn2*-deficient mice display a decrease in pain sensitivity and a slight decrease in motor co-ordination, and, most importantly, a marked increase in anxiety-like behavior.^37, 38^

So far cortical circuit organization has been studied predominantly in sensory cortices such as the visual and somatosensory cortices.^39, 40^ Despite the presence of an increasing amount of experimental evidences that emphasizes the importance of the dichotomy of the PFC and its connections with the amygdala for regulating fear behavior,^4, 5, 6^ we still know very little about the fear-related synaptic circuit within the PFC.^17^ How circuits are organized in agranular cortices like the PFC, which lacks a granular L4 layer, and how GABAergic neurons that play a vital role in neural circuitry are distributed in this area, is even less known.^8, 41, 42, 43^ Therefore, detailed knowledge about the involving GABAergic neuronal circuit between IL and PrL could become a key link in our understanding of physiology and pathophysiology in the central regulation of fear and anxiety behaviors.

To the best of our knowledge, there are so far no published data demonstrating a direct inhibitory synaptic connection between IL and PrL.^4, 5, 6^ By convention, most cortical GABAergic neurons are referred to as GABAergic interneurons, as they typically project a highly ramified axon to neurons in their close vicinity. On the other hand, it has been shown that a small population of these GABAergic neurons can also give rise to long-range cortico-cortical projections. To distinguish them from GABAergic interneurons, this class of GABAergic neurons is referred to as GABAergic projection neurons, although their functional relevance in the cortical network is still at issue.^44^ These GABAergic projection neurons have been suggested to account for only 0.5% of the whole population of GABAergic neurons and part of them have been assumed to be NPY^+^-GABAergic neurons.^44^ In the present study, we hypothesized that pyramidal neurons of IL might activate NPY^+^-GABAergic neurons and directly inhibit the pyramidal neurons in the ipsilateral PrL. In this way, IL would not only lead to an activation of the downstream central nucleus of the amygdala,^12, 13^ but also lead to inhibition of the ipsilateral PrL, resulting in reduced activation of the basal amygdala nucleus and finally an anxiolytic effect.^14, 15^ In addition, we investigated whether deletion of *Nlgn2* would influence inhibitory transmission from IL to PrL, and thus may be causally involved in increased anxiety-like behaviors that have been reported in these mice.^37, 38^

## Materials and methods

Detailed information about immunohistochemistry and electrophysiology is provided in Supplementary Information.

### Animals

All experiments were performed in accordance with the European Communities Council Directive (86/EEC), and were approved by the State Office for Nature, Environment and Consumer Protection of North Rhine-Westphalia, Germany (LANUV NRW). For immunohistochemistry and electrophysiology, adult male wild-type C57BL/6 mice, transgenic PV–eGFP mice,^45^ NPY–eGFP mice^46^ and *Nlgn*2-KO mice^34^ (10–16 weeks old) were used.

### Immunohistochemistry

Coronal sections of 25 μm in thickness were cut using cryostat (Leica CM3050 S, Leica Microsystems Nussloch, Nussloch, Germany) from the PFC of the brains (from 1.98 to 1.54 mm anterior to Bregma) for immunohistochemistry and immunofluorescent procedures. For detection of PV and NPY, standard immunohistochemistry staining procedures were performed (details provided in Supplementary Information) with two different anti-PV (P3088 and SAB4200545; Sigma-Aldrich, St. Louis, MO, USA; both 1:500) as well as anti-NPY (ab10980; Abcam, Cambridge, UK; 1:500) as primary antibodies. For fluorescence imaging, tissues were visualized using an epifluorescent IX81 microscope (Olympus, Münster, Germany) and for confocal imaging a 700-AX10 laser scanning microscope (Carl Zeiss, Jena, Germany) was used.

### Quantification analysis and Image acquisition

For quantification, the brain areas and the layer borders were defined according to the mouse brain atlas^47^ and based on cytoarchitectural features as described before.^9, 48, 49^ Multiple alignment of images taken with × 4 and × 10 magnification was performed with Cell^P software (Olympus). Distributions of positively stained cells were analyzed using ImageJ software (NIH, Bethesda, MD, USA) in the anterior cingulated cortex (ACC), PrL, IL and motor cortex 2 (M2). For each region, mean numbers of cells as well as cell density (cell × mm^−2^) were calculated across all the layers in above regions. For better comparison, the mean numbers of cells have also been calculated as cell × mm^−3^ that were presented in Supplementary Information (Supplementary Tables 4 and 5). For NPY^+^-GABAergic neurons, the layer specific distribution of different subtypes was calculated as percentage of total numbers (Figure 3).

### Electrophysiology

All recordings were performed in neurons of PFC (Schema see Figure 4a) as described before (details in Supplementary Information).^50, 51^ Spontaneous GABAergic inhibitory postsynaptic currents (sIPSCs) were recorded at a holding potential of −70 mV in the presence of the 10 μM AMPA-receptor antagonist 6-cyano-7-nitroquinoxaline-2,3-dione (CNQX), 50 μM NMDA-receptor antagonist 2-amino-5-phosphono-valeric acid (AP5) and 2 μM glycine receptor antagonist strychnine. For assessing neuronal firing properties, square current pulses (first level −50 pA, increment 10 pA, duration 500 ms) were injected from a holding level corresponding to −70 mV holding potential every 5 s. For each neuron, several parameters were estimated (details in Supplementary Information). For further fluorescent marking, the pipette solution was supplemented with biocytin (1mg ml^−1^; Sigma-Aldrich).

### Data analysis

Data were presented as mean±s.e.m. with number of cells per animals indicated in parentheses. Student's *t*-test or non-parametric Mann–Whitney test were used to determine differences between data samples for normally- and non-normally distributed data, respectively. Statistical significance is indicated as * for *P*<0.05, ** for *P*<0.01, *** for *P*<0.001.

## Results

### PV^+^-GABAergic neurons are differentially distributed in ACC, PrL and IL

**Figure 1:**
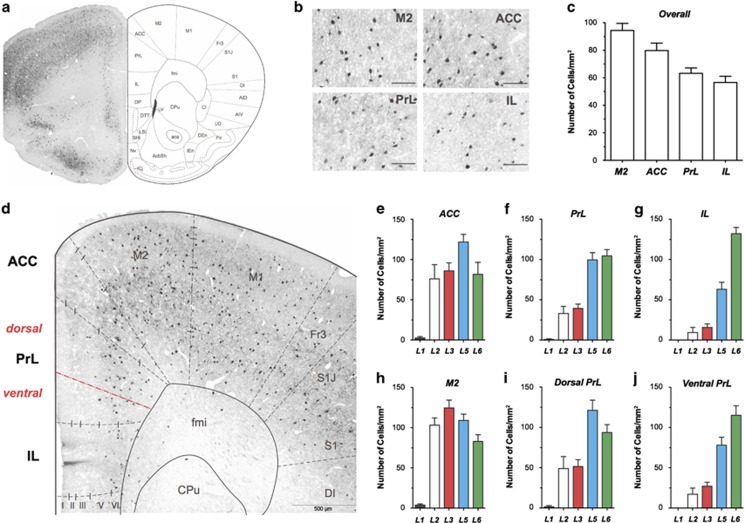
The distribution of PV^+^-GABAergic neurons is different in M2, ACC, PrL and IL. (**a**) Overview of PV-stained coronal sections of PFC (left) in 1.70 mm anterior to Bregma and the schematic drawing of different areas; (**b**) The shapes of PV^+^-GABAergic neurons were quite similar; (**c**) Quantification of the overall density of PV^+^-GABAergic neurons in M2, ACC, PrL and IL; (**d**) PV^+^−stained coronal sections of PFC in higher magnification. (**e**–**j**) Layer-specific quantification of the density of PV^+^-GABAergic neurons in ACC (**e**), PrL (**f**), IL (**g**), M2 (**h**), dorsal (**i**) and ventral parts (**j**) of PrL. ACC, anterior cingulated cortex; IL, infralimbic cortex; M2, motor cortex 2; PrL, prelimbic cortex.

Immunoreactivity of PV was present across different tested brain areas although their distribution patterns were different (Figure 1a). Higher-magnification images showed that the somata of PV^+^-GABAergic neurons were round and the dendritic arborizations were multipolar in all tested brain areas (Figure 1b). On average, the densities of PV^+^-GABAergic neurons were 94.4±5.2 mm^−2^, 79.8±5.3 mm^−2^, 64.3±4.3 mm^−2^ and 56.6±4.5 mm^−2^ for M2, ACC, PrL and IL, respectively (Figure 1c).

It is quite striking that there were very few PV^+^-GABAergic cells in layer II and III of PrL and IL (Figures 1a and d–j; details see also Supplementary Table 1). The distribution of PV^+^-GABAergic neurons was not homogeneous in PrL (Figure 1d) with the dorsal part being similar to M2 and ACC, but the ventral part being similar to IL. Further quantification supported these results (Figures 1e and j; details see also Supplementary Table 1). Staining with another independent antibody for PV confirmed the above mentioned results (Supplementary Figure 1).

### The distribution of NPY^+^-GABAergic neurons is different in ACC, PrL and IL

**Figure 2:**
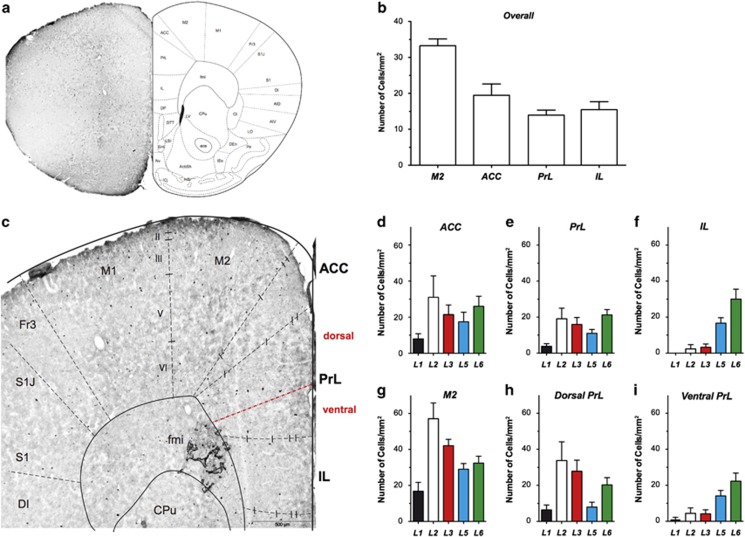
The distribution of NPY^+^-GABAergic neurons is different in ACC, PrL and IL. (**a**) Overview of NPY-stained coronal sections of PFC (left) in 1.70 mm anterior to Bregma and the schematic drawing of different areas. (**b**) Quantification of the overall density of NPY^+^-GABAergic neurons in M2, ACC, PrL and IL. (**c**) NPY^+^−stained coronal sections of PFC in higher magnification. (**d**–**i**) Layer-specific quantification of the density of NPY^+^-GABAergic neurons in ACC (**d**), PrL (**e**) and IL (**f**), M2 (**g**), dorsal (**h**) and ventral parts (**i**) of PrL. ACC, anterior cingulated cortex; IL, infralimbic cortex; M2, motor cortex 2; PrL, prelimbic cortex.

Immunoreactivity of NPY was present across different tested brain areas although their distribution was quite sparse in some regions. The total numbers of NPY^+^-GABAergic neurons were about three times smaller as compared with PV^+^-GABAergic neurons (Figure 2a). On average, the densities of NPY^+^-GABAergic neurons were 33.3±1.9 mm^−2^ in M2, whereas they were 19.5±3.1 mm^−2^, 14.0±1.4 mm^−2^ and 15.5±2.2 mm^−2^ for ACC, PrL and IL, respectively (Figure 2b).

Besides the lower overall densities of NPY^+^-GABAergic cells, it is quite striking that they were almost absent in layers I–III of IL (Figures 2a and c–g; Supplementary Table 2). In addition, the distribution of NPY^+^-GABAergic neurons in the dorsal part of PrL was similar to M2 and ACC, but in the ventral part of PrL being similar to IL, thus closely resembling the distribution pattern of PV^+^-GABAergic neurons (*cf.*, Figure 1). Further quantification again supported these findings with similar densities of NPY^+^-GABAergic neurons in the dorsal PrL, the M2 and the ACC region (Figures 2d; Supplementary Table 2). In contrast, the ventral PrL again resembled the IL (Figures 2f and i; Supplementary Table 2).

### There are three main classes of NPY^+^-GABAergic neurons

**Figure 3:**
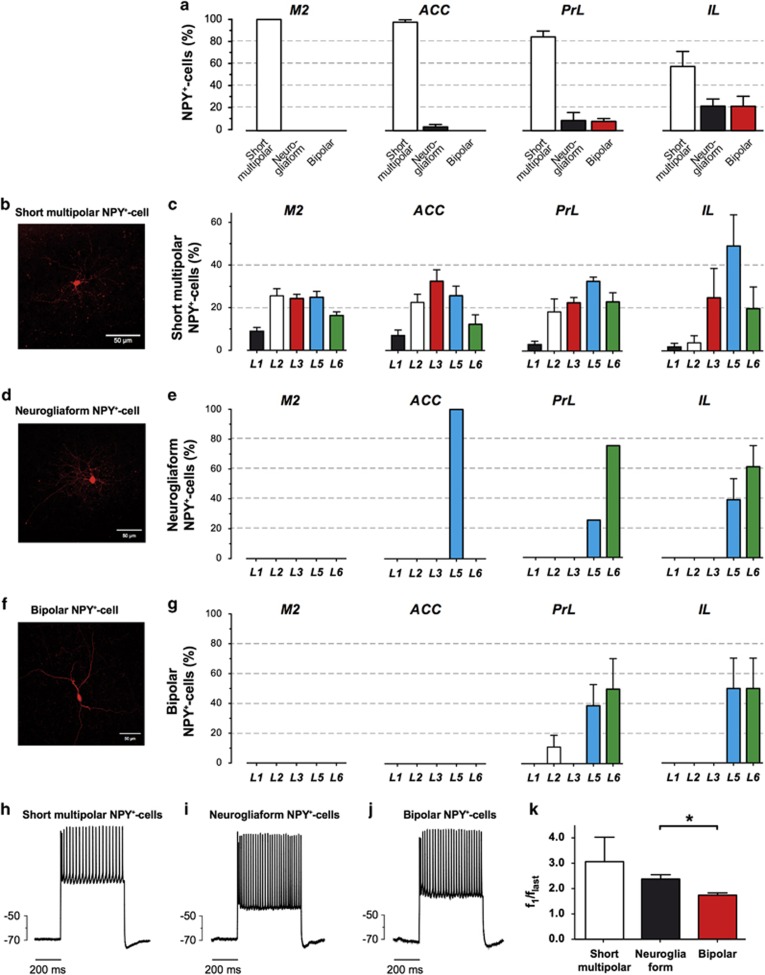
The distribution of three different subtypes of NPY^+^-GABAergic neurons in ACC, PrL, IL and M2. (**a**) The three subtypes were calculated as mean percentage of total NPY^+^-GABAergic neurons for each region. (**b**) Example of a short process multipolar NPY^+^-GABAergic neuron. (**c**) Layer-specific distribution in M2, ACC, PrL and IL as percentage of total numbers of short process multipolar NPY^+^-GABAergic neurons in the corresponding areas. (**d**) Example of a neurogliaform NPY^+^-GABAergic neuron. (**e**) Layer-specific distribution in M2, ACC, PrL and IL as percentage of total numbers of neurogliaform NPY^+^-GABAergic neurons in the corresponding areas. (**f**) Example of a bipolar NPY^+^-GABAergic neuron. (**g**) Layer-specific distribution in M2, ACC, PrL and IL as percentage of total numbers of bipolar NPY^+^-GABAergic neurons in the corresponding areas. (**h-j**) Train of spikes during a 500-ms depolarization pulse in a short process multipolar cell (**h**); a neurogliaform cell (**i**) and a bipolar cell (**j**). (**k**) Spike adaptation ratio *f*_1_/*f*_last_ of neurogliaform, bipolar and multipolar neurons. *P<0.05. ACC, anterior cingulated cortex; IL, infralimbic cortex; M2, motor cortex 2; PrL, prelimbic cortex.

In all tested areas, morphologically examined NPY^+^-GABAergic neurons could be categorized into three main classes: (i) short process multipolar^52, 53^ (Figure 3b); (ii) neurogliaform^22, 52, 54^ (Figure 3d) and (iii) bipolar^52, 55^ (Figure 3f). Short multipolar NPY^+^-GABAergic cells were the most abundant form in M2 and PFC (Figure 3a; 100% in M2 to 58% in IL), Neurogliaform and bipolar NPY^+^-GABAergic cells were overall less abundant, but accumulated in ventral regions of the PFC, especially in PrL (up to 8% of NPY^+^-GABAergic cells; Figure 3a) and most abundantly in IL (21% Figure 3a). It is interesting to note that the latter two subtypes were nearly exclusively located in output layers V and VI of PrL and IL (>90% Figures 3e and g).

By injection of incremental depolarizing currents to the NPY–eGFP neurons, we observed appearance of the action potential (spike), which then developed into a train of spikes of increasing frequency up to a certain maximum (other electrophysiological properties see Supplementary Table 3). All examined NPY^+^ neurons were fast-spiking (average frequency ~60 Hz) with mild frequency adaptation within 500 ms (Figures 3h and j). In bipolar cells the adaptation ratio *f*_1_/*f*_last_ appeared to be significantly lower than that of neurogliaform cells in PrL and IL (*P*=0.03), while short multipolar cells in M2 had higher adaptation ratio similar to that of neurogliaform cells (Figure 3k). Bipolar cells also tended to show higher excitability, although the differences in these values did not reach the level of statistical significance (Supplementary Table 3).

### The pyramidal neurons of PL receive a direct inhibitory input from ipsilateral IL

**Figure 4:**
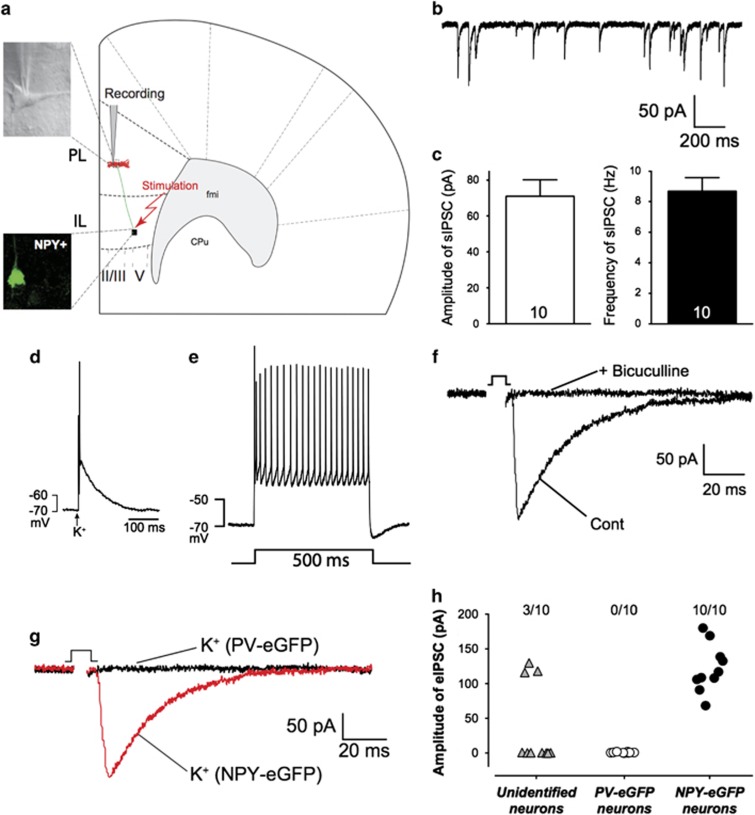
The pyramidal neurons of PrL receive a direct inhibitory input from NPY^+^-GABAergic projection neurons of ipsilateral IL. (**a**) Schematic drawing of the position of stimulation and recording electrodes; (**b** and **c**) Sample traces (**b**), amplitude and frequency of spontaneous IPSCs in layer II/III pyramidal neurons of PrL (**c**); (**d**) Sample trace of an action potential in GABAergic interneuron elicited by 1-ms K^+^-application; (**e**) Spike train in a small unidentified neuron during 500-ms-long current injection in current-clamp mode; (**f**) iontophoretic activation of unidentified small neurons in layer V of IL evoked bicuculline-sensitive IPSC in pyramidal neurons of PrL; (**g**) Examples of IPSCs evoked in pyramidal neurons by iontophoretic activation of NPY–eGFP neurons in IL (red trace); absence of responses on stimulation of PV–eGFP neurons in IL (black trace); (**h**) Summary of all experimental recordings of eIPSCs in layer II pyramidal neurons of PrL. Note each data point illustrated an individual experiment, while the numbers over each column indicated the success rate of eliciting eIPSCs (*n*/*N*: *n*=successful experiments, *N*=total number of experiments). IL, infralimbic cortex; IPSC, inhibitory postsynaptic current; PrL, prelimbic cortex.

Recordings of spontaneous electrical activity indicated that layer II pyramidal neurons in PrL received strong inhibitory inputs (averaged sIPSC amplitude: 70.9±8.3pA; frequency 8.8±0.9 Hz; *n*=10; Figures 4b and c). Further characterization provided first evidence of a direct GABAergic input from the ipsilateral IL, as bicuculline-sensitive IPSCs could be evoked in PrL pyramidal neurons by extracellular stimulation in layer V of the ipsilateral IL (eIPSC amplitude: 71±29 pA; Figures 4a and f).

We next tested whether the inhibitory response evoked from layer V of IL in pyramidal neurons in layer II of PrL (Figure 4g) might be mediated by GABAergic projection neurons by recording of layer II pyramidal neurons in PrL, while the stimulation electrode (glass pipette, tip~2 μM; containing ACSF+100 mM K^+^) was placed directly on the soma of a small unidentified neuron in layers V–VI of ipsilateral IL (schema, Figure 4a). Iontophoretic activation by K^+^ (1 ms) elicited a single action potential (Figure 4d), when such unidentified cells were patch-clamped, a rectangular current injection evoked a fast-spiking pattern, typical for interneurons (Figure 4e). In this way, iontophoretic activation of a subset (Figure 4h, left panel) of small neurons by K^+^ evoked a bicuculline-sensitive IPSC in pyramidal neurons of PrL, indicating that the inhibitory input from IL to PrL was directly mediated by GABAergic neurons in ipsilateral IL.

### The inhibitory input from IL to PrL is mediated by NPY^+^-GABAergic projection neurons in IL

It is interesting that no eIPSC could be elicited in pyramidal neurons of PrL when PV–eGFP neurons^45^ in layer V of IL were iontophoretically stimulated (Figures 4g and h, middle panel). On the other hand, iontophoretic activation of NPY–eGFP neurons^46^ in IL by K^+^, when the electrode was placed directly on the soma of the eGFP neurons, indeed evoked bicuculline-sensitive IPSCs (Figures 4g and h, right panel), while no responses could be elicited in pyramidal neurons of PrL when the electrode was placed beside the soma of this neuron in IL (data not shown). These evoked bicuculline-sensitive IPSCs could be recorded in pyramidal neurons of both dorsal and ventral part of PrL. It is also noteworthy that the amplitudes of eIPSC evoked by stimulation of unidentified neurons and by NPY–eGFP neurons were quite similar (Figure 4h). Thus, our data suggested that the pyramidal neurons in layers II/III of both dorsal and ventral part of PrL received direct inhibitory input mediated by NPY^+^-GABAergic projection neurons in ipsilateral IL.

### Deletion of Nlgn2 attenuates the inhibitory input from IL to PrL

**Figure 5:**
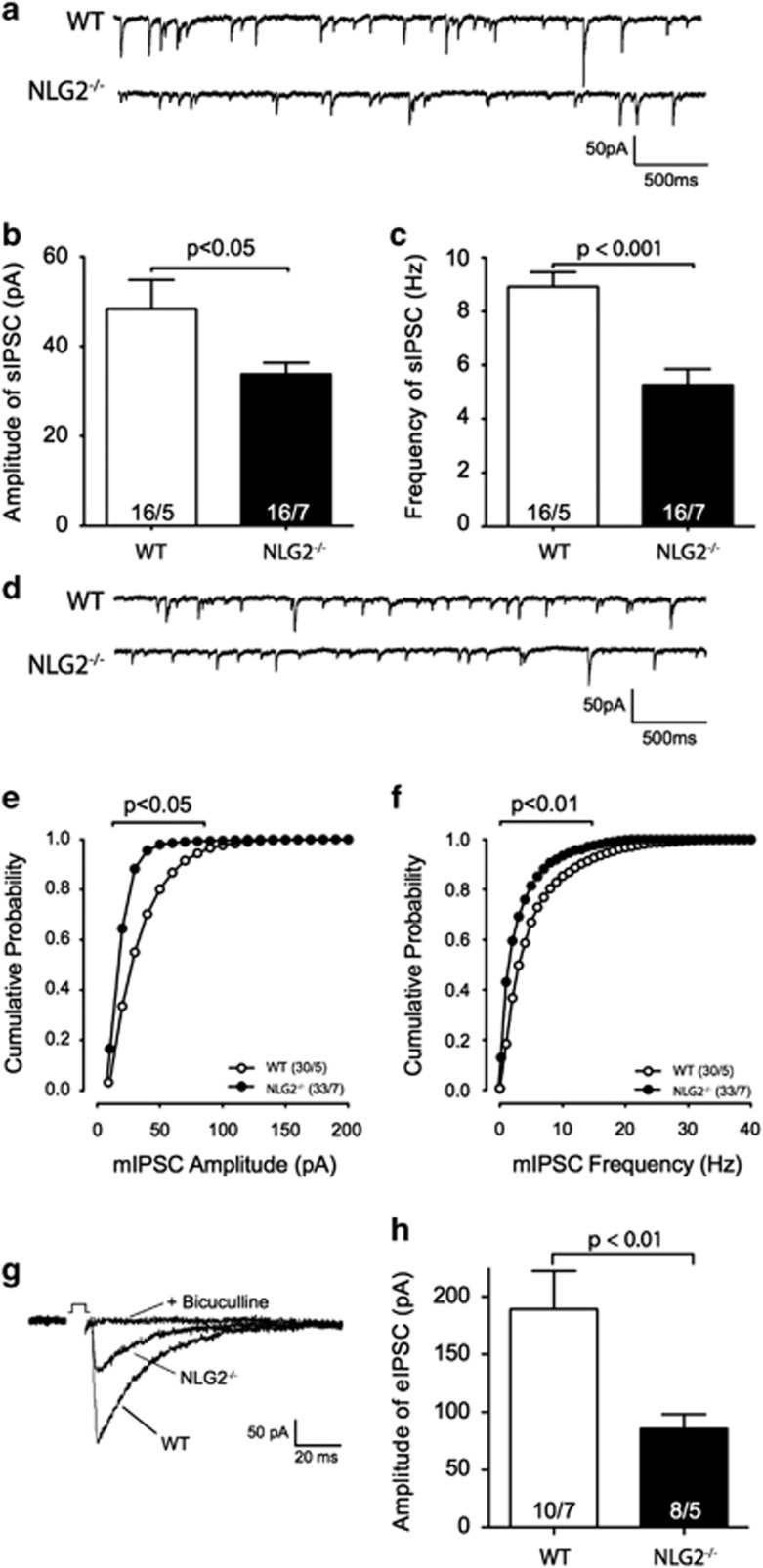
Deletion of Nlgn2 attenuates the overall inhibitory inputs to PrL, especially the synaptic inhibition from IL to PrL. (**a–c**) Sample traces (**a**), averaged amplitude (**b**) and averaged frequency (**c**) of spontaneous IPSCs in pyramidal neurons of PrL; (**d–f**) Sample traces (**d**), cumulative probability of amplitude (**e**) and frequency (**f**) of miniature IPSCs in pyramidal neurons of PrL; (**g** and **h**) Sample traces (**g**) and average amplitudes (**h**) of IPSCs in layer II/III pyramidal neurons of PrL evoked by stimulation in IL in wild-type (WT) and *Nlgn2*^−/−^ mice. Note that the numbers within the bar diagrams *n/N* indicate the number of cells (*n*) tested/number of mice (*N*). IL, infralimbic cortex; IPSC, inhibitory postsynaptic current; PrL, prelimbic cortex.

*Nlgn2*-defficient mice display a marked increase in anxiety-like behavior as compared with their wild-type littermates.^37, 38^ We therefore ask the question whether deficiency of *Nlgn2* would influence inhibitory transmission from IL to PrL. Indeed, the spontaneous inhibitory transmission in PrL was significantly reduced in *Nlgn2*-KO mice compared with their WT littermates (Figures 5a and c, sIPSC amplitude: 48.4±6.5 pA in WT, *n*=16/5; 33.8±2.5 pA in *Nlgn2*-KO, *n*=16/7; *P*<0.05; sIPSC frequency: 8.9±0.5 Hz in WT, *n*=16/5; 5.3±0.6 Hz in Nlgn2-KO, *n*=16/7; *P*<0.001). The miniature IPSCs in PrL were significantly attenuated in *Nlgn2*-KO mice (Figures 5d and f, mIPSC amplitude: 34.3±1.5 pA in WT, *n*=30/5; 28.2±1.4 pA in *Nlgn2*-KO, *n*=33/7; *P*<0.05; mIPSC frequency: 7.6±0.5 Hz in WT, *n*=30/5; 4.5±0.4 Hz in *Nlgn2*-KO, *n*=33/7; *P*<0.01). In addition, iontophoretically evoked bicuculline-sensitive IPSCs from layer V of IL to pyramidal neurons in layer II of ipsilateral PrL were also significantly reduced in *Nlgn2*-KO mice as compared with their wild-type littermates (Figures 5g and h: eIPSC amplitude: 189±33 pA in WT, *n*=10/7; 86±13 pA in *Nlgn2*-KO, *n*=8/5; *P*<0.01). Thus these data indicate that deletion of *Nlgn2*-gene causes a significant attenuation of overall inhibitory inputs to pyramidal neurons in PrL as well as the direct inhibition from IL to PrL.

## Discussion

The present study provided five novel findings: (1) the distribution of PV^+^- and NPY^+^-GABAergic neurons was different in PrL and IL as compared with M2 and ACC; (2) the PrL could be divided in a dorsal and a ventral part; (3) IL directly inhibited the ipsilateral pyramidal neurons of both dorsal and ventral part of PrL; (4) this direct inhibition was not mediated by PV^+^-, but by NPY^+^-GABAergic projection neurons in IL; (5) deletion of *Nlgn2* caused significant attenuation of the inhibitory transmission from IL to PrL. Given the importance of the dichotomic network between IL and PrL in central control of emotion, activation of IL would suppress the activation of PrL-related pathways and thus differentially change the activation of downstream limbic areas and subsequently shape the fear expression and fear extinction both in physiology and pathophysiology.

### Inhibitory neuronal organization in PFC

In the present study, the distribution patterns of PV and NPY were similar between ACC and M2 (Figures 1 and 2). This pattern can be therefore referred to as a common 'cortical type'. On the other hand, the density of PV^+^- and NPY^+^-neurons was very low in layer II and III of PrL and IL (Figures 1). They can thus be referred to as a 'prefrontal type'. Further detailed analysis revealed that, regarding to the distribution pattern of PV^+^− and NPY^+^−neurons, the PFC can be divided into a 'cortical type' comprising ACC and dorsal PrL and a 'prefrontal type' comprising ventral PrL and IL.

To comprehend the functional consequences of the above data, it would be worthwhile to investigate the distribution and the specific projections of other GABAergic neurons, such as calretinin-, somatostatin-positive GABAergic neurons, which are at present unexplored.^8, 41, 42, 43^ This would be especially interesting for layers II/III, where the expression of PV^+^- and NPY^+^-neurons was very rare. Not only will it be interesting to know which subtypes of GABAergic neurons are expressed in layer II/III of PrL and IL, also the interconnectivity between these neurons and the pyramidal neurons is largely unknown. Furthermore, molecular analysis on the single cell level^56, 57^ will greatly contribute to our understanding of neuronal circuits in the PFC.

Although we do not know the functional consequences yet, we want to suggest dividing the PrL in two parts: the 'cortical type' PrL_dorsal_ and the 'prefrontal type' PrL_ventral_. This classification might be premature yet in the absence of a clear functional understanding of their contribution to the neocortical network. However, it is quite likely that both parts are interconnected with different brain areas with different neuronal oscillatory features and therefore need quite different equipped GABAergic neuronal circuits.

### Neuronal circuit between IL and PrL

The PFC has extensive connections with the subcortical limbic areas and thalamus^2, 3^ and has been functionally implicated in processes of emotional regulation.^4, 5, 6^ The dichotomic effects of IL and PrL on fear expression are mediated by their outputs to different targets within the amygdala.^1, 16, 17^ In addition, non-amygdala outputs of IL and PrL are also emerging as important targets for emotional regulation.^58, 59^ Our present data provide the first experimental evidence that IL and PrL might reciprocally regulate the activity of each other, such that the activation of IL leads to direct inhibition of pyramidal neurons in PrL (Figure 4).

By convention cortical glutamatergic neurons are considered the sole originators of long-range projections, while cortical GABAergic interneurons are typically described as only projecting their axons locally.^44^ Previous data revealed that a subset of GABAergic neurons also project axons to remote neocortical regions.^60, 61, 62, 63^ In addition, it has been shown that microstimulation of the neocortex elicits monosynaptic inhibitory postsynaptic potentials in the remote ipsilateral cortex.^64, 65^ It has therefore been proposed that neurons in different cortical areas may need to be connected reciprocally and symmetrically via GABAergic projection neurons for synchronization of gamma-oscillations in multiple cortical areas,^66^ although their functional significance has remained uncertain.^44^ In the present study, we showed for the first time that IL provides direct inhibitory input to ipsilateral ventral and dorsal PrL by activation of NPY^+^-GABAergic projection neurons in IL (Figure 4). This data fits very well to the observation that most cortical GABAergic projection neurons show immunoreactivity of somatostatin, NPY or nNOS.^44^ Thus, activation of these NPY^+^-GABAergic projection neurons in IL will lead to direct inhibition of pyramidal neurons in ipsilateral PrL and thus attenuate the activation of its downstream targets. One would thus expect that activation of the NPY^+^-GABAergic projection neuron-mediated inhibition would alleviate the activation of PrL-related anxiogenic pathway, and accentuate the IL-related anxiolytic pathway.^1, 16, 17^ To comprehend the neuronal circuits between PrL and IL, it would be worthwhile to further investigate that the synaptic inhibition originates from GABAergic neurons located in PrL that reciprocally inhibit the pyramidal neurons in IL.

### The role of Nlgn2 in the neuronal circuit between IL and PrL

Consistent with its localization *in vivo,*^31^ Nlgn2 appears to function primarily at inhibitory synapses.^33^ Furthermore, our previous findings demonstrated that Nlgn2 binds the scaffolding protein gephyrin and functions as a specific activator of collybistin, thus guiding the clustering of inhibitory neurotransmitter receptors. Deletion of *Nlgn2* perturbs GABAergic and glycinergic synaptic transmissions and leads to a loss of postsynaptic specializations.^33, 34, 35, 36, 37^ As a consequence of this, *Nlgn2*-KO mice demonstrate heightened anxiety-related behavior on multiple measures.^38^ Given the broad expression of Nlgn2 at inhibitory synapses throughout the brain,^31^ this selective anxiety-related phenotype is somehow surprising. One possible explanation is that the loss of Nlgn2 could be partially compensated by other Nlgn isoforms in many brain regions. However, there was no compensatory increase in Nlgn1 or Nlgn3 levels in *Nlgn2*-KO mice.^38^ As Nlgn2 is differentially expressed in different brain areas,^31^ the functional roles of Nlgn2-related control of maturation of inhibitory synapses in PFC and limbic areas might be more predominant than in other brain areas. Indeed, our present result showed that deletion of *Nlgn2*-gene could not be compensated in PFC and therefore significantly diminished the overall GABAergic inhibitory inputs to PrL, especially the inhibition from IL to ipsilateral PL (Figure 5). These dismantled inhibitions to PrL would lead to an unbalanced accentuation of PrL-related activation of downstream limbic areas, and thus bias the fear regulation in favor of increased anxiety-like behavior as shown previously.^37, 38^

Taken together, we propose that the dichotomic neuronal circuit in PFC does not only contain the PrL-related excitatory circuit and the IL-related inhibitory circuit including their downstream target areas,^1, 16, 67^ but it must also include the neuronal circuit between IL and PrL that reciprocally regulates the activity of each other, although many details about the related neuronal circuits are still elusive. Our present results provide the first experimental evidence for the existence of such a direct inhibition from IL to ipsilateral PrL. Within this reciprocal neuronal circuit between IL and PrL, the inhibitory input from PrL to IL will predominate during fear expression/renewal, while the inhibitory input from IL to PrL predominates during fear extinction/extinction recall. In this way, fear extinction results not only from increased activity within the IL-related inhibitory circuit, but it also occurs via simultaneous decreased activity of the PrL-related excitatory circuit mediated by NPY^+^-GABAergic projection neurons in IL. Given the existence of the reciprocal inhibitions between IL and PrL, this simultaneous deactivation of PrL may be necessary to additionally remove the possible reversal inhibition from PrL to IL and to further facilitate the IL-related extinction learning.

## Conclusion

Future studies are necessary to determine the detailed synaptic interconnectivities within this reciprocal neuronal circuit between IL and PrL. Furthermore, it is important to consider the exact subtypes of GABAergic neurons that are studied. Indeed, for several neuromodulators, it is already known that they are co-localized in a subtype-specific way.^52, 68^ A detailed knowledge of the role of neuromodulators within the neuronal circuits throughout the different layers of the PFC could lead to a deepened understanding of neuronal processing under various physiological and pathophysiological conditions.
