# Supplementary figures and images for: NPY+-, but not PV+-GABAergic neurons mediated long-range inhibition from infra- to prelimbic cortex
Source: Transl Psychiatry. 2016 Feb 16;6(2):e736–. doi: 10.1038/tp.2016.7 (PMC4872436; doi:10.1038/tp.2016.7)

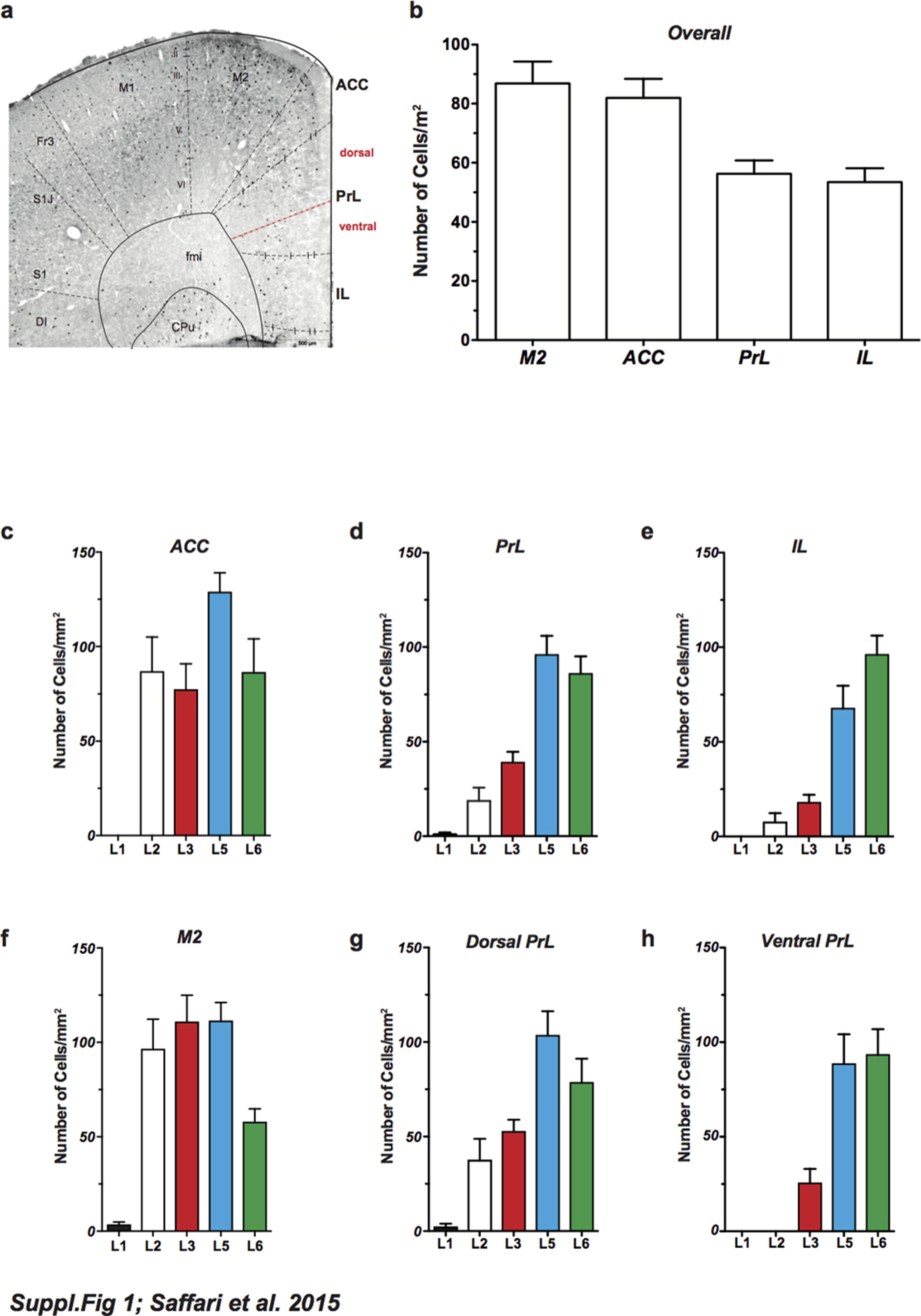

Supplement: Supplementary Figure 1 [file tp20167x1.tif]
