# Supplementary material for: NPY+-, but not PV+-GABAergic neurons mediated long-range inhibition from infra- to prelimbic cortex
Source: Transl Psychiatry. 2016 Feb 16;6(2):e736–. doi: 10.1038/tp.2016.7 (PMC4872436; doi:10.1038/tp.2016.7)
Supplement: Supplementary Table 1 [file tp20167x2.docx]

| ***Supplemental Table 1*** | | | | | | | | | | | | | | | | |
| --- | --- | --- | --- | --- | --- | --- | --- | --- | --- | --- | --- | --- | --- | --- | --- | --- |
| Densities (cell/mm^2^) of PV^+^-GABAergic neurons in different layers of mPFC and M2. | | | | | | | | | | | | | | | | |
|  |  |  | | **Layer** | | | | | | | | | | | | |
| ***Area*** |  | **I** |  | |  | **II** |  |  | **III** |  |  | **V** |  |  | **VI** |  |
| ***M2*** | 3.8 | ± | 1.5 | | 103.2 | ± | 9.0 | 124.5 | ± | 9.8 | 109.1 | ± | 7.8 | 83.0 | ± | 8.4 |
| ***ACC*** | 2.5 | ± | 1.7 | | 76.0 | ± | 17.8 | 85.9 | ± | 9.9 | 121.8 | ± | 9.5 | 81.7 | ± | 14.9 |
| ***PrL_total_*** | 0.8 | ± | 0.8 | | 33.1 | ± | 8.7 | 39.3 | ± | 5.4 | 99.6 | ± | 8.9 | 104.5 | ± | 7.8 |
| ***IL*** |  | - |  | | 9.3 | ± | 6.4 | 15.7 | ± | 4.4 | 63.1 | ± | 8.7 | 132.1 | ± | 7.7 |
| ***PrL_dorsal_*** | 1.5 | ± | 1.5 | | 49.0 | ± | 14.8 | 51.4 | ± | 8.5 | 121.2 | ± | 12.6 | 93.7 | ± | 9.7 |
| ***PrL_ventral_*** |  | - |  | | 17.2 | ± | 7.7 | 27.1 | ± | 5.0 | 78.1 | ± | 9.9 | 115.3 | ± | 11.8 |
