# Supplementary material for: NPY+-, but not PV+-GABAergic neurons mediated long-range inhibition from infra- to prelimbic cortex
Source: Transl Psychiatry. 2016 Feb 16;6(2):e736–. doi: 10.1038/tp.2016.7 (PMC4872436; doi:10.1038/tp.2016.7)
Supplement: Supplementary Table 2 [file tp20167x3.docx]

| ***Supplemental Table 2*** | | | | | | | | | | | | | | | |
| --- | --- | --- | --- | --- | --- | --- | --- | --- | --- | --- | --- | --- | --- | --- | --- |
| Densities (cell/mm^2^) of NPY^+^-GABAergic neurons in different layers of mPFC and M2. | | | | | | | | | | | | | | | |
|  |  | **Layer** | | | | | | | | | | | | | |
| **Area** |  | **I** |  |  | **II** |  |  | **III** |  |  | **V** |  |  | **VI** |  |
| ***M2*** | 16.8 | ± | 4.9 | 57.1 | ± | 8.7 | 42.1 | ± | 3.4 | 29 | ± | 3.1 | 32.4 | ± | 3.8 |
| ***ACC*** | 8 | ± | 2.9 | 31 | ± | 12 | 21.4 | ± | 5.4 | 17.5 | ± | 5.3 | 26.1 | ± | 5.5 |
| ***PrL_total_*** | 3.8 | ± | 1.5 | 19.1 | ± | 5.9 | 16 | ± | 3.8 | 11 | ± | 2.1 | 21.3 | ± | 3 |
| ***IL*** |  | - |  | 2.4 | ± | 2.3 | 3.3 | ± | 1.8 | 16.6 | ± | 3 | 30 | ± | 5.5 |
| ***PrL_dorsal_*** | 6.4 | ± | 2.7 | 34 | ± | 10 | 27.8 | ± | 6.2 | 8 | ± | 2.7 | 20.3 | ± | 4 |
| ***PrL_ventral_*** | 1.1 | ± | 1.1 | 4.4 | ± | 3 | 4.1 | ± | 2.2 | 14.1 | ± | 3 | 22.2 | ± | 4.5 |
