# Supplementary material for: NPY+-, but not PV+-GABAergic neurons mediated long-range inhibition from infra- to prelimbic cortex
Source: Transl Psychiatry. 2016 Feb 16;6(2):e736–. doi: 10.1038/tp.2016.7 (PMC4872436; doi:10.1038/tp.2016.7)
Supplement: Supplementary Table 3 [file tp20167x4.docx]

| ***Supplemental Table 3*** |  |  |  |  |  |  |  |
| --- | --- | --- | --- | --- | --- | --- | --- |
| Passive, excitability and firing properties of NPY^+^-neurons in PFC | | | | | | | |
|  | Neuroglia (n=17) | | | Bipolar (n=17) | | | *P* |
| RMP (mV) | -60 | ± | 1.9 | -56 | ± | 6.7 | 0.39 |
| Input resistance (MΩ) | 284 | ± | 30 | 403 | ± | 88 | 0.37 |
| Membrane capacitance (pF) | 86 | ± | 22 | 75 | ± | 7.3 | 0.66 |
| Time constant (ms) | 21 | ± | 3.2 | 31 | ± | 8.8 | 0.23 |
| Averaged frequency (Hz) | 65 | ± | 5 | 59 | ± | 8.7 | 0.59 |
| Rheobase (pA) | 94 | ± | 8.4 | 67 | ± | 13 | 0.08 |
| f_1_ (Hz) | 135 | ± | 11 | 105 | ± | 17 | 0.11 |
| f_2_ (Hz) | 104 | ± | 14 | 80 | ± | 15 | 0.46 |
| f_last_ (Hz) | 57 | ± | 3.7 | 60 | ± | 7.7 | 0.79 |
| AP threshold (mV) | -38 | ± | 1.8 | -39 | ± | 1.0 | 0.73 |
| Maximal spike number | 31 | ± | 2.4 | 30 | ± | 4.3 | 0.84 |
| Spike amplitude (mV) | 51 | ± | 2.8 | 65 | ± | 11 | 0.29 |
| AHP (mV) | -15 | ± | 1.4 | -16 | ± | 2.0 | 0.61 |
| Half-width (ms) | 1.5 | ± | 0.12 | 1.3 | ± | 0.16 | 0.43 |
| Depolarization slope (mV/ms) | 122 | ± | 10 | 171 | ± | 41 | 0.17 |
| Repolarization slope (mV/ms) | -42 | ± | 3.8 | -55 | ± | 10 | 0.34 |
