# Supplementary material for: NPY+-, but not PV+-GABAergic neurons mediated long-range inhibition from infra- to prelimbic cortex
Source: Transl Psychiatry. 2016 Feb 16;6(2):e736–. doi: 10.1038/tp.2016.7 (PMC4872436; doi:10.1038/tp.2016.7)
Supplement: Supplementary Table 4 [file tp20167x5.doc]

| ***Supplemental Table 4*** | | | | | | | | | | | | | | | | |
| --- | --- | --- | --- | --- | --- | --- | --- | --- | --- | --- | --- | --- | --- | --- | --- | --- |
| Densities (cell/mm^3^) of PV^+^-GABAergic neurons in different layers of mPFC and M2. | | | | | | | | | | | | | | | | |
|  |  |  | | **Layer** | | | | | | | | | | | | |
| ***Area*** |  | **I** |  | |  | **II** |  |  | **III** |  |  | **V** |  |  | **VI** |  |
| ***M2*** | 153 | ± | 58 | | 4130 | ± | 360 | 4980 | ± | 390 | 4360 | ± | 310 | 3320 | ± | 340 |
| ***ACC*** | 98 | ± | 67 | | 3040 | ± | 710 | 3440 | ± | 400 | 4870 | ± | 380 | 3270 | ± | 590 |
| ***PrL_total_*** | 30 | ± | 30 | | 1320 | ± | 350 | 1570 | ± | 210 | 3990 | ± | 350 | 4180 | ± | 310 |
| ***IL*** |  | - |  | | 370 | ± | 260 | 630 | ± | 170 | 2520 | ± | 350 | 5280 | ± | 310 |
| ***PrL_dorsal_*** | 60 | ± | 60 | | 1960 | ± | 590 | 2060 | ± | 340 | 4850 | ± | 500 | 3750 | ± | 390 |
| ***PrL_ventral_*** |  | - |  | | 690 | ± | 310 | 1090 | ± | 200 | 3120 | ± | 390 | 4610 | ± | 470 |
