# Supplementary material for: NPY+-, but not PV+-GABAergic neurons mediated long-range inhibition from infra- to prelimbic cortex
Source: Transl Psychiatry. 2016 Feb 16;6(2):e736–. doi: 10.1038/tp.2016.7 (PMC4872436; doi:10.1038/tp.2016.7)
Supplement: Supplementary Table 5 [file tp20167x6.doc]

| ***Supplemental Table 5*** | | | | | | | | | | | | | | | |
| --- | --- | --- | --- | --- | --- | --- | --- | --- | --- | --- | --- | --- | --- | --- | --- |
| Densities (cell/mm^3^) of NPY^+^-GABAergic neurons in different layers of mPFC and M2. | | | | | | | | | | | | | | | |
|  |  | **Layer** | | | | | | | | | | | | | |
| **Area** |  | **I** |  |  | **II** |  |  | **III** |  |  | **V** |  |  | **VI** |  |
| ***M2*** | 670 | ± | 200 | 2280 | ± | 350 | 1690 | ± | 140 | 1160 | ± | 130 | 1300 | ± | 150 |
| ***ACC*** | 320 | ± | 110 | 1240 | ± | 480 | 860 | ± | 210 | 700 | ± | 210 | 1040 | ± | 220 |
| ***PrL_total_*** | 150 | ± | 60 | 760 | ± | 240 | 640 | ± | 150 | 440 | ± | 80 | 850 | ± | 120 |
| ***IL*** |  | - |  | 90 | ± | 90 | 130 | ± | 70 | 670 | ± | 120 | 1200 | ± | 220 |
| ***PrL_dorsal_*** | 260 | ± | 110 | 1350 | ± | 410 | 1110 | ± | 250 | 320 | ± | 110 | 810 | ± | 160 |
| ***PrL_ventral_*** | 40 | ± | 40 | 180 | ± | 120 | 170 | ± | 90 | 560 | ± | 120 | 890 | ± | 180 |
